# Supplementary material for: Gene Coexpression Network Analysis Indicates that Hub Genes Related to Photosynthesis and Starch Synthesis Modulate Salt Stress Tolerance in Ulmus pumila
Source: Int J Mol Sci. 2021 Apr 23;22(9):4410. doi: 10.3390/ijms22094410 (PMC8122946; doi:10.3390/ijms22094410)
Supplement: Supplementary file 1 [file ijms-22-04410-s001.zip › SUPP/Figure S1-S3.docx]

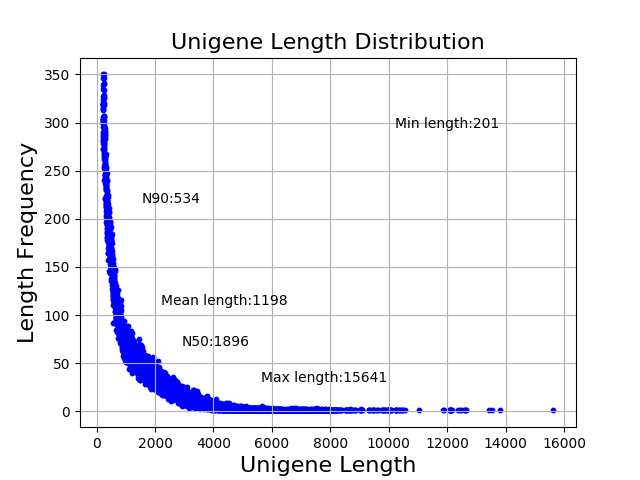


**Figure S1.** Unigene length Distribution. The vertical axis means the length frequency of assembled unigenes, and the horizontal axis means the length of assembled unigenes.

**
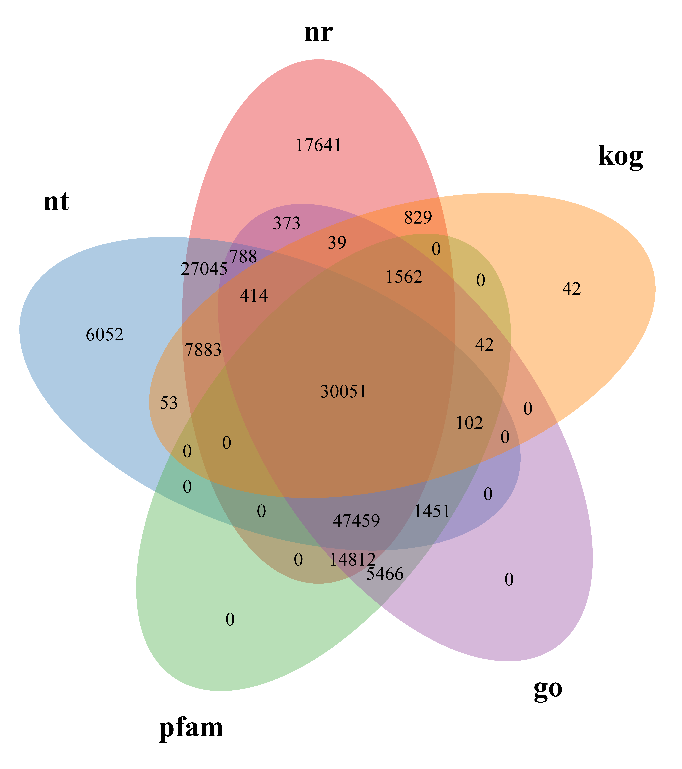
**

**Figure S2.** Gene functional annotation. a. Veen diagram of assembled unigenes in five nucleic acid or protein database, including nucleic acid or Protein database (nr), Nucleic acid or protein database (nt), Protein family (pfam), Gene Ontology (go), and Clusters of Orthologous Groups of proteins (kog).


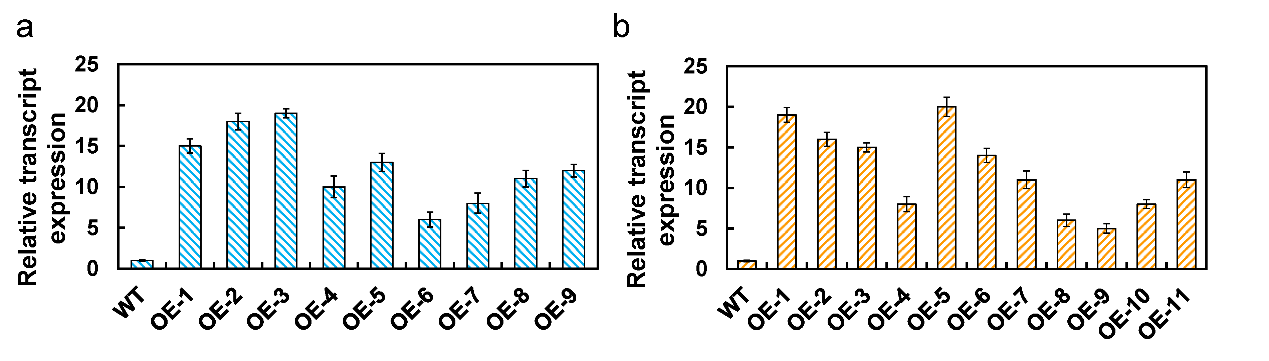


**Figure S3 Relative expression levels of two hub genes in transgenic and wild-type Arabiodpsis detected by RT-qPCR.** (a) *UpPETH* and (b) *UpWAXY*. Data are presented as means ± SD (n = 3).
