# Supplementary figures and images for: Gene Coexpression Network Analysis Indicates that Hub Genes Related to Photosynthesis and Starch Synthesis Modulate Salt Stress Tolerance in Ulmus pumila
Source: Int J Mol Sci. 2021 Apr 23;22(9):4410. doi: 10.3390/ijms22094410 (PMC8122946; doi:10.3390/ijms22094410)

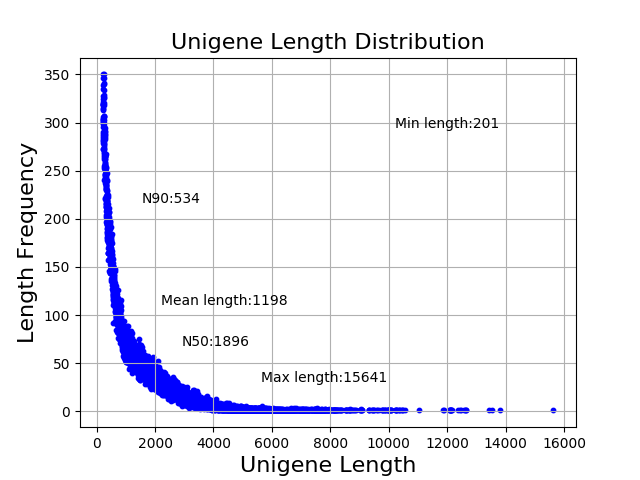

Supplement: Supplementary file 1 [file ijms-22-04410-s001.zip › SUPP/TIFF/Additional Figure S1.tif]

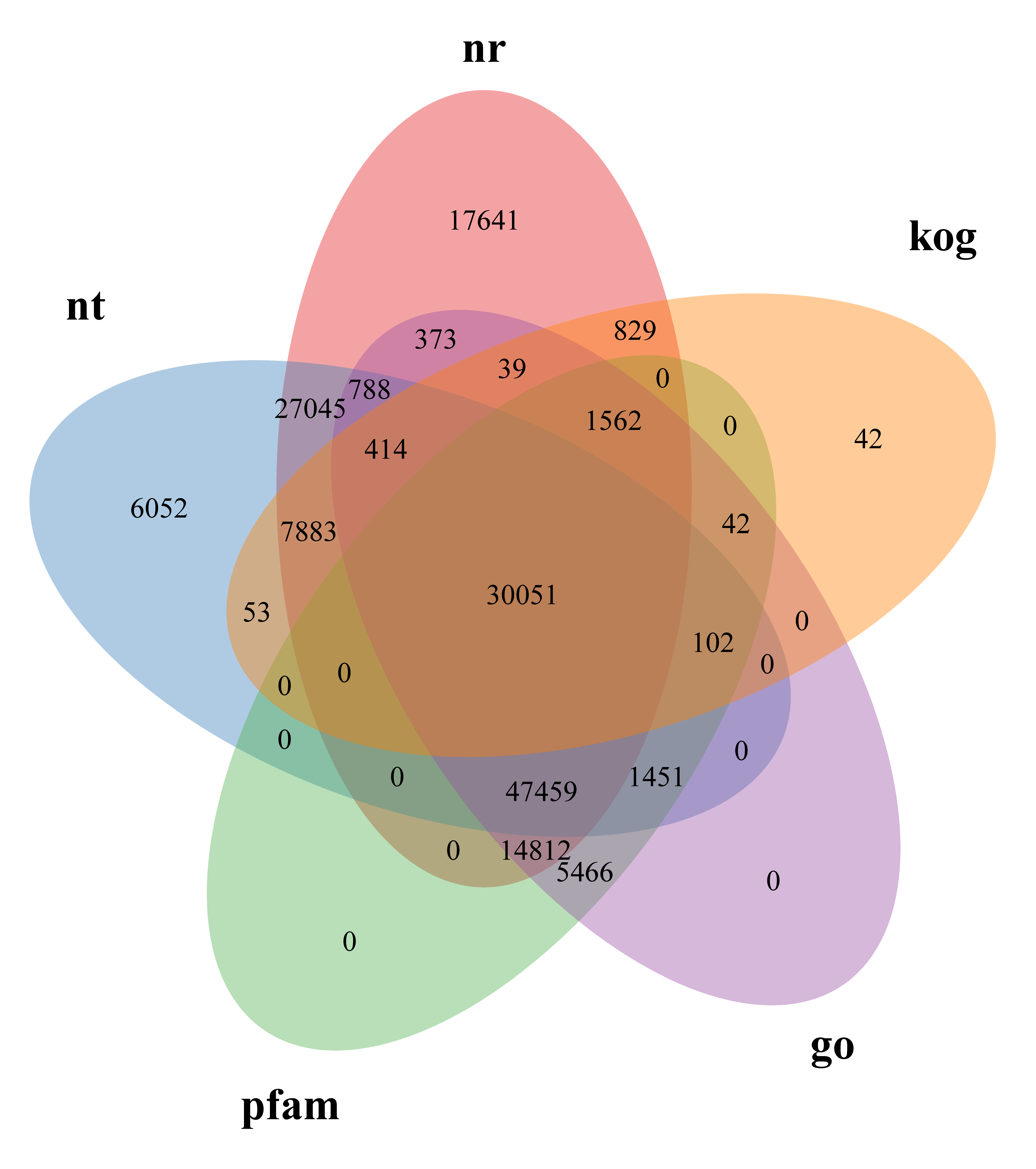

Supplement: Supplementary file 1 [file ijms-22-04410-s001.zip › SUPP/TIFF/Additional Figure S2.tif]

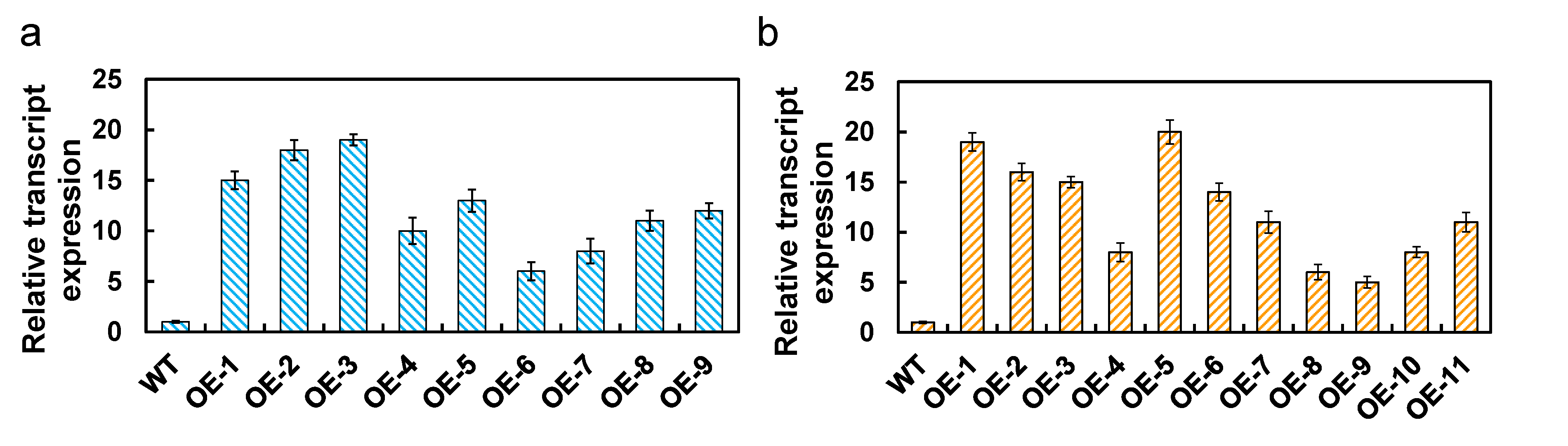

Supplement: Supplementary file 1 [file ijms-22-04410-s001.zip › SUPP/TIFF/Additional Figure S3.tif]
